# Detection of ureaplasmas and bacterial vaginosis associated bacteria and their association with non-gonococcal urethritis in men

**DOI:** 10.1371/journal.pone.0214425

**Published:** 2019-04-04

**Authors:** Maria Frølund, Lars Falk, Peter Ahrens, Jørgen Skov Jensen

**Affiliations:** 1 Research Unit for Reproductive Microbiology, Statens Serum Institut, Copenhagen S, Denmark; 2 Department of Dermatovenereology, Linköping University Hospital and Linköping University, Linköping, Sweden; Fred Hutchinson Cancer Research Center, UNITED STATES

## Abstract

No aetiology is found in up to 40% of men with symptomatic urethritis. Male partners of women with bacterial vaginosis (BV) may be at higher risk of non-gonococcal urethritis (NGU). The aim of this study was to examine the role of BV associated bacteria in first-void urine (FVU) in 97 asymptomatic men without urethritis (controls) and 44 men (cases) with NGU including 20 men with idiopathic urethritis (IU) attending a Swedish STD-clinic between January and October 2010. BV-associated bacteria and ureaplasmas were detected by quantitative PCR assays. All BV associated bacteria, except *Megasphaera*-like type 1, were strongly positively correlated with *U*. *urealyticum* p<0.005 and even stronger with the combined *U*. *urealyticum* and *U*. *parvum* load (p<0.0005) suggesting that ureaplasma induced elevated pH may stimulate the growth of BV associated bacteria. No statistically significant differences were found between IU cases and controls in the prevalence or load of BV associated bacteria or ureaplasmas. In multiple logistic regression, *Megasphaera*-like type 1 was associated with IU (p = 0.03), but most positive FVU samples contained very few bacteria and the finding may not be clinically relevant.

## Introduction

Symptoms of urethritis is a common reason for attendance at sexually transmitted diseases (STD) clinics and in most cases, urethritis is caused by sexually transmitted pathogens [1]. In Scandinavia, non-gonococcal urethritis (NGU) is much more common than urethritis caused by *Neisseria gonorrhoeae*, although there has been a steady increase of reported cases of *N*. *gonorrhoeae* in the last decade from 7 to 25 cases per 100,000 inhabitants in Sweden [2, 3]. NGU is characterised by inflammation of the male urethra and symptoms such as urethral discharge and dysuria are common. However, asymptomatic urethritis with microscopically documented inflammation on stained urethral smears is frequent and well documented for *Chlamydia trachomatis*, *Mycoplasma genitalium* and *Ureaplasma urealyticum* [4–6]. If left untreated, *C*. *trachomatis* infection and probably *M*. *genitalium* infection may be transmitted to female partners and cause pelvic inflammatory disease and infertility. No aetiology is found in the up to 40% of male urethritis cases where known pathogens such as *C*. *trachomatis*, *M*. *genitalium*, *N*. *gonorrhoeae*, *Trichomonas vaginalis*, *U*. *urealyticum*, herpes simplex virus type 1 and 2, and adenovirus have been excluded [7, 8].

It has been suggested that male partners to women with bacterial vaginosis (BV) are at a higher risk of NGU [1, 9, 10], but this has not been widely studied. BV, present in 10–20% of Scandinavian women [11, 12], is the most common vaginal infection in women with symptoms such as abnormal vaginal discharge, foul odour, itching and burning, although many women have no symptoms or have been accustomed to the condition [13]. It has been associated with a higher risk of preterm birth, HIV transmission, acquisition of other STDs and pelvic inflammatory disease [14, 15]. BV is characterized as an imbalance of the normal bacterial microbiota in the vagina where lactobacilli are reduced and replaced by anaerobic bacteria, the most frequently *Gardnerella vaginalis*, *Atopobium vaginae*, *Prevotella* spp., *Eggerthella*-like uncultured bacteria, *Megasphaera*-like type 1, *Sneathia* spp. and Bacterial Vaginosis Associated Bacterium– 2 (BVAB-2) [16].

However, little is known about the presence of BV associated bacteria in men and their association with NGU. One study found that male partners to women with BV were more likely to suffer from NGU [17] and a more recent study showed one of the key BV associated species *Sneathia amnii* (previously *Leptotrichia amnionii*) or *S*. *sanguinegens* to be associated with male NGU [10]. In men, first void urine has been shown to be an excellent proxy for the urethral microbiota [18] and since this sample type is non-invasive, this is the preferred sample for studies of the urethral microbiota. Next generation sequencing methods and sequencing the 16S rRNA gene in urine have shown that BV associated bacteria such as *Prevotella* spp., *Gardnerella* spp., *Mobiluncus* spp, *Veillonella* spp., *Atopobium* spp. and *Megaspherea* spp. are found in both sexually experienced and inexperienced men [19, 20].

The aim of this study was to examine the role of selected BV associated bacteria: *G*. *vaginalis*, BVAB-2, *Eggerthella*-like uncultured bacteria, *Megasphaera*-like type 1, *S*. *amnii*, *A*. *vaginae*, *S*. *sanguinegens* and *Prevotella* spp as well as *U*. *parvum* in urine specimens from men with and without NGU attending an STD clinic. These species were selected based on their strong association with BV in a previous study [21, 22] and because *U*. *parvum* is found very frequently in the female lower genital tract; however, the role of this species is still disputed [23].

## Materials and methods

### Study design

The study was a cross-sectional case-control study in men attending a Swedish STD-clinic.

### Patients and specimens

Men attending the STD clinic in Norrköping, Sweden were included in this study as they took part in a cross-sectional study of prevalence, signs and symptoms of various bacterial STIs among 614 men. Patients selected for the present study were enrolled between January 2010 and October 2010. Men without symptoms and signs of urethritis by microscopy serving as controls in this study, attended the clinic for a check-up for STIs (42%) or as part of contact tracing of patients with STIs (55%); 2% attended for erectile dysfunction. Cases and controls negative for *C*. *trachomatis* and *M*. *genitalium* were consecutively selected for the present study. In addition, the first nine *C*. *trachomatis* positive and the first nine *M*. *genitalium* cases, all negative for *U*. *urealyticum* and *U*. *parvum* and still fulfilling the inclusion criteria were selected aiming to control for associations with BV associated bacteria in men with known causes of urethritis. All selected samples were analysed by polymerase chain reaction (PCR) for adenovirus [6, 24], HSV 1 and 2 and selected BV associated bacteria [22].

Data were collected on a standard questionnaire regarding the reasons for attendance, age, symptoms of urethritis (dysuria and discharge), number of sexual partners within the last year and life-time sexual partners, condom use at the first sexual intercourse with a new partner, sexual intercourse with men (MSM), previous STIs, antibiotic treatment within the last month, current partner (yes or no), whether the partner had a current STI and if so which STI. The patients’ identity was coded and the variables noted in a database.

Urethral smears were obtained from all patients with a blunt curette and stained with methylene blue. All smears were examined with a Zeiss (standard 14) light microscope by the physician. Patients with dysuria and/or discharge and smears showing ≥5 polymorphonuclear leukocytes (PMNL) per high-power (×1,000) microscopic field (hpf) in more than 4 hpfs were defined as having urethritis. Low grade urethritis was defined as 5–9 PMNL/hpf, Medium grade as 10–30 PMNL/hpf, and high grade as >30 PMNL/hpf. The controls had no symptoms of urethritis and had urethral smears with <5 PMNL/hpf. They had no other symptoms or signs of genital disease such as ulcers, itching, warts or rash. Neither cases nor controls had received antibiotics within the previous month prior to enrolment.

When *N*. *gonorrhoeae* was suspected by microscopy (intracellular diplococci) or due to high-risk behaviour, culture was performed, and if positive, the patient was excluded. The patients had not voided within at least one hour before sampling. After the clinical examination, the patient was asked to collect approximately 20 mL of first void urine; 7–13 mL was immediately decanted into 1.1 mL Genelock transport medium (Sierra Molecular Corp., Sonoma, CA, USA) and sent the same day by mail to Statens Serum Institut (SSI), Copenhagen, Denmark. Specimens were received and DNA extracted in the laboratory within a median of 5 days (mean 4.5 days; range 2–22 days) after they had been collected. From the remaining urine, 3 mL was collected in a Multi-collect tube (Abbott, IL, USA) and analysed at the Linköping University Hospital laboratory within two days for *C*. *trachomatis* using the Abbott m2000 system. DNA was extracted from 400 μL according to the manufacturer’s protocol. This assay employs two separate targets of the cryptic plasmid detecting also the new variant *C*. *trachomatis*, which is common in Sweden and described initially in 2007 [25]. *Trichomonas vaginalis* was not included in the screening panel as this organism is extremely rare in the population under study.

### PCR assays

All other PCR assays were performed at SSI, Copenhagen. Urine samples in Genelock medium were concentrated by centrifugation of 1,900 μL at 30,000×*g* for 15 min. The pellet was resuspended in 300 μL of a 20% Chelex-100 slurry (BioRad, Hercules, CA) in TE-buffer (10 mM Tris-HCL [pH 8.0], 1 mM EDTA) and incubated at 95° for 10 minutes as previously described [26].The prepared samples were stored at –20°C until PCR was performed.

*M*. *genitalium* was detected by real-time PCR targeting the MgPa-gene [26] and confirmed by conventional PCR targeting the 16S rRNA gene [27]. Macrolide resistance-mediating mutations were demonstrated by sequencing region V of the 23S rRNA gene in *M*. *genitalium* positive samples [28]. For detection of *U*. *urealyticum* and *U*. *parvum*, a multiplex PCR targeting the urease gene was used [29].

HSV 1 and 2 were detected with a multiplex real-time Taqman based PCR assay [6]. The primers produced a <100-basepair fragment of the Envelope Glycoprotein G and D in HSV 1 and 2, respectively. Target specific HSV 1 and 2 probes were labelled with FAM and VIC, respectively. Adenovirus was detected with real-time PCR amplifying the hexon gene using the primerset AdF1 and AdR1 [24].

Detection of the BV associated bacteria *G*. *vaginalis*, BVAB-2, *Eggerthella*-like uncultured bacteria, *Megasphaera*-like type 1, *S*. *amnii*, *A*. *vaginae*, *S*. *sanguinegens* and *Prevotella* sp. was performed by qPCR as previously described [6, 22]. These species were selected based on their strong association with BV [21, 22]. All qPCR results were expressed as DNA copies per mL of the original urine sample.

## Ethics statement

Men attending the STD clinic in Norrköping, Sweden were included in this study as they took part in a cross-sectional study of prevalence, signs and symptoms of various bacterial STIs among 614 men. Patients selected for the present study were enrolled between January 2010 and October 2010. The study was approved by the regional research ethical committee of Linköping (protocol # M 134–09). All men received written and oral information about the study and all provided written informed consent.

All samples were coded and analysed anonymously in the SSI research laboratory. Routine testing for STIs at Linköping University Hospital laboratory was performed without anonymization according to standard procedures.

### Statistical analysis

Fisher´s exact test and odds-ratios with confidence intervals were used for dichotomous variables, the Mann-Whitney test for continuous variables, Spearman’s correlation coefficients and multiple logistic regression and other statistical analyses were calculated in StatsDirect (StatsDirect Ltd., Cheshire, UK).

Graphs were produced in GraphPad Prism (GraphPad Software, Inc., La Jolla, CA, USA).

Significance level was 5%, and two-sided results were used throughout.

## Results

### Characteristics of included patients

**Fig 1:**
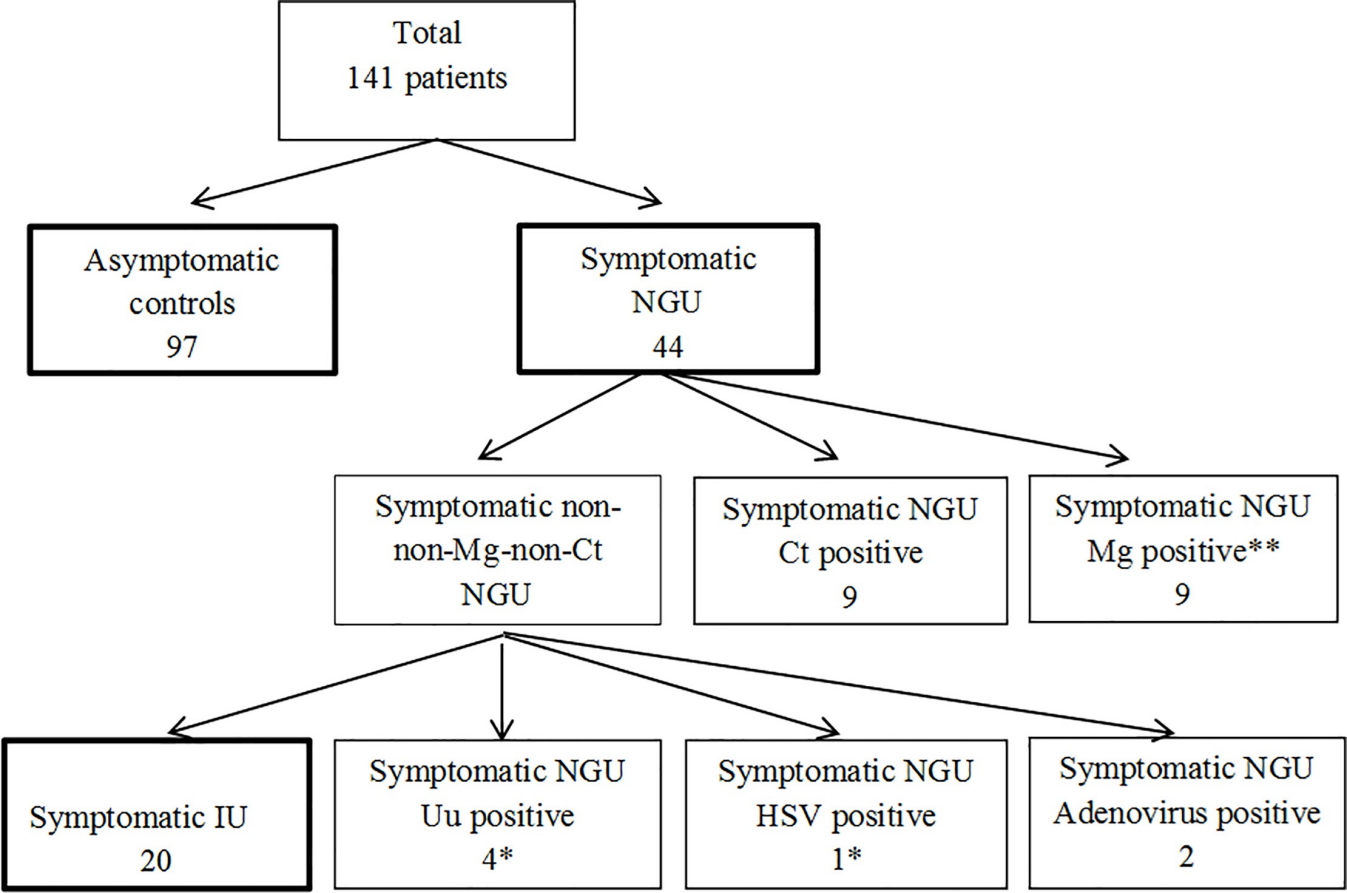
Flowchart showing included male patients (n = 141) and the different subgroups. *C*. *trachomatis* (Ct), *M*. *genitalium* (Mg), *U*. *urealyticum* (Uu), Idiopatic urethritis (IU).* 26 samples were *U*. *urealyticum* positive. †One sample was double-infected with HSV type 1 and *M*. *genitalium*.^‡^ One sample was double-infected with HSV type 1 and *U*. *urealyticum*.

A total of 141 patients were included of which 97 men were asymptomatic controls and 44 were symptomatic cases. In the latter group, 20 men were negative for *C*. *trachomatis*, *M*. *genitalium*, HSV, adenovirus and *U*. *urealyticum* and defined as having idiopathic urethritis (IU) (Fig 1).

Controls (n = 97), patients with IU (n = 20) and NGU patients with known aetiology (n = 24) did not differ with respect to median age, number of life-time sexual partners or partners within the last year, MSM or with respect to habits for condom use at first sexual intercourse with a new partner (Table 1).

**Table 1:** Characteristics of patients (n = 141) in the groups “Asymptomatic controls”, “Symptomatic NGU with known aetiology” and “Symptomatic IU”.

Characteristic	Asymptomatic controls (n = 97)	Symptomatic NGU with known aetiology (n = 24)	Symptomatic IU (n = 20)
Age in years	25 (23–30)	25 (20–41)	24 (22–30)
No. sex partners, past year	3 (2–4)*	3 (1–20)	4 (3–6)
No. life-time sex partners	13 (8–23)	17 (7–100)	16 (12–25)^
MSM	2 (2.1%)^	0 (0.0%)	0 (0.0%)
Regular sex partner	55 (56.7%)	11 (45.8%)	11 (55%)
Condom use^^			
*Usually*	32 (33.0%)	6 (25.0%)	5 (25.0%)
*Never or rarely*	49 (50.5%)	15 (62.5%)	13 (65.0%)
*Always first intercourse*	11 (11.3%)	3 (12.5%)	2 (10.0%)
Symptoms			
*Dysuria*	-	20 (83.3%)	18 (90.0%)
*Discharge*	-	13 (54.2%)	10 (50.0%)
*Urethral itching*	-	0 (0.0%)	3 (15.0%)
PMNL/hpf			
*<5*	97	-	-
*5–10*	-	4 (16.7%)	6 (30.0%)
*10–30*	-	20 (83.3%)	13 (65.0%)
*>30*	-	0 (0.0%)	1 (5.0%)

N (%) for dichotomous data. Median (range) for continuous, non-normally distributed variables (all). None of the comparisons reached a 5% level of significance.

* No information for 3 persons

^ No information for 1 person

^^ Condom use at first sexual intercourse with a new partner (No information for 5 persons in the group of asymptomatic controls (5.2%))

The distribution of symptoms in the patients with IU was similar to that of the 24 patients with urethritis with known aetiology for dysuria, discharge and urethral itching 18 (90%) vs. 20 (83%), 10 (50%) vs. 13 (54%) and 3 (15%) vs. 0 (0%), respectively. Neither did the distribution of low, medium, or high-grade urethritis differ between cases with IU and patients with NGU of known aetiology (Table 1).

### Distribution of NGU associated organisms

Of the 97 asymptomatic controls, 26 men (27%) were *U*. *urealyticum* positive. When excluding the nine *C*. *trachomatis* and nine *M*. *genitalium* positives, the remaining non-*M*. *genitalium*, non-*C*. *trachomatis* symptomatic NGU (NMGNCNGU) cases comprised 26 patients (cases) (Fig 1). In this group, four men (15%) were *U*. *urealyticum* positive. No difference in *U*. *urealyticum* prevalence (positive rate) between controls and cases (p = 0.31) or in bacterial load could be demonstrated (p = 0.31). Twenty-five (26%) controls were *U*. *parvum* positive compared with six men in the NMGNCNGU group (23%) (NS). No difference in bacterial load could be demonstrated for *U*. *parvum*.

None of the controls were HSV or adenovirus positive. One control was co-infected with *U*. *urealyticum* and *U*. *parvum*. Among 26 NMGNCNGU cases, one man (4%) was HSV-type 1 positive (1,250 copies/mL and co-infected with *U*. *urealyticum*) and two men were adenovirus positive (type D and type F, respectively). Adenovirus was found significantly more frequently among the NMGNCNGU cases (p = 0.04).

### Association between *U*. *parvum* and BV associated bacteria in IU compared with controls

*U*. *parvum* was analysed together with the BV associated bacteria as it was not considered a cause of NMGNCNGU whereas *U*. *urealyticum* was excluded from the IU group. None of the BV associated bacteria or *U*. *parvum* were found more frequently in the group of symptomatic IU cases (n = 20) than in controls (n = 97) in univariate analysis. The median DNA load for BV associated bacteria and *U*. *parvum* did not differ between the controls and the patients with IU (Table 2). There was a trend towards *Megasphaera*-like type 1 being present more often and in higher loads among men with IU than in controls (p = 0.09 and p = 0.1, respectively).

**Table 2:** Bacterial vaginosis associated bacteria and *Ureaplasma parvum* in asymptomatic male controls (n = 97) and in men with symptomatic idiopathic urethritis (n = 20). Symptomatic IU patients are negative for *C*. *trachomati*s, *M*. *genitalium*, HSV, adenovirus and *U*. *urealyticum*.

Organism	Asymptomatic controls	Symptomatic IU	Crude OR (95% CI)	P
	(n = 97)	(n = 20)		
	Bacterial load (DNA copies/mL)*			
***U*. *parvum***				
Positive n (%)	25 (26)	6 (30)	1.2 (0.35–3.89)	0.78
Median (range)	0 (0–170,625)	0 (0–10,050)	…	0.75
***G*. *vaginalis***				
Positive n (%)	69 (71)	11 (55)	0.50 (0.17–1.52)	0.19
Median (range)	250 (0–3,028,500)	69 (0–314,500)	…	0.1
**BVAB2**				
Positive n (%)	21 (22)	3 (15)	0.64 (0.11–2.54)	0.76
Median (range)	0 (0–643,000)	0 (0–964,125)	…	0.74
***Eggerthella*-like bacterium**				
Positive n (%)	30 (31)	4 (20)	0.56 (0.13–1.94)	0.42
Median (range)	0 (0–22,339,125)	0 (0–93,250)	…	0.25
***Megasphaera*-like type 1**				
Positive n (%)	13 (13)	6 (30)	2.77 (0.73–9.45)	0.09
Median (range)	0 (0–38)	0 (0–125)	…	0.1
***S*. *amnii***				
Positive n (%)	19 (20)	4 (20)	1.03 (0.22–3.71)	>0.99
Median (range)	0 (0–929,125)	0 (0–33,750)	…	0.97
***A*. *vaginae***				
Positive n (%)	36 (37)	6 (30)	0.73 (0.21–2.24)	0.62
Median (range)	0 (0–301,375)	0 (0–16,750)	…	0.43
***S*. *sanguinegens***				
Positive n (%)	19 (20)	3 (15)	0.72 (0.12–2.91)	0.76
Median (range)	0 (0–6,190,009)	0 (0–155,125)	…	0.72
***Prevotella* spp.**				
Positive n (%)	68 (70)	16 (80)	1.71 (0.49–7.59)	0.43
Median (range)	2,000 (0–6,943,000)	688 (0–182,211,625)	…	0.84

*original urine sample

The presence of *U*. *parvum* and BV associated bacteria was analysed in a multivariate model adjusting for all tested pathogens (Table 3). *Megasphaera*-like type 1 was associated with urethritis with adjusted odds ratios (ORadj) 4.6 (95% CI 1.2–18.6) (*p* = 0.03).

**Table 3:** Adjusted odds ratios (ORadj) with 95% confidence intervals (CI) and p-values in asymptomatic male controls (n = 97) and in men with symptomatic idiopathic urethritis (n = 20) after multivariate logistic regression analysis for eight bacterial vaginosis associated bacteria and *Ureaplasma parvum*.

	OR_adj_	95% CI	p-value
*U*. *parvum*	1.7	0.5 to 5.7	p = 0.38
*G*. *vaginalis*	0.4	0.1 to 1.3	p = 0.13
BVAB2	0.5	0.1 to 3.1	p = 0.48
*Eggerthella*-like bacterium	0.9	0.2 to 4.4	p = 0.88
*Megasphaera*-like type 1	4.6	1.2 to 18.3	p = 0.03
*S*. *amnii*	1.3	0.3 to 5.9	p = 0.72
*Prevotella spp*	2.1	0.66 to 7.8	p = 0.24
*A*. *vaginae*	0.7	0.2 to 3.0	p = 0.60
*S*. *sanguinegens*	0.7	0.1 to 3.9	p = 0.72

*G*. *vaginalis* and *Prevotella* spp. were the BV associated bacteria most commonly found, and they were detected in both patient groups. Seventy-one percent of the controls and 55% of the IU cases were *G*. *vaginalis* positive, whereas 70% and 80% respectively were *Prevotella* spp. positive (NS) (Table 2).

### Relationship between urethral inflammation and ureaplasmas and BV associated bacteria

The bacterial load of ureaplasmas and BV associated bacteria was analysed according to the stratified urethral inflammatory response (<5; 5–10; >10 PMNL/hpf) for all men included in the study regardless of the cause of urethritis. Only 10 men had low-grade urethritis (5–10 PMNL/hpf) but the combined *U*. *urealyticum* and *U*. *parvum* load was significantly lower (p = 0.009) in this group than in the asymptomatic controls. The only other significant difference was seen for *G*. *vaginalis* which was found to have a higher median load in controls than in men with high grade urethritis (p = 0.03) suggesting a possible suppression of this species by the inflammatory response.

### Co-occurence between BV associated bacteria and ureaplasma in all samples

**Fig 2:**
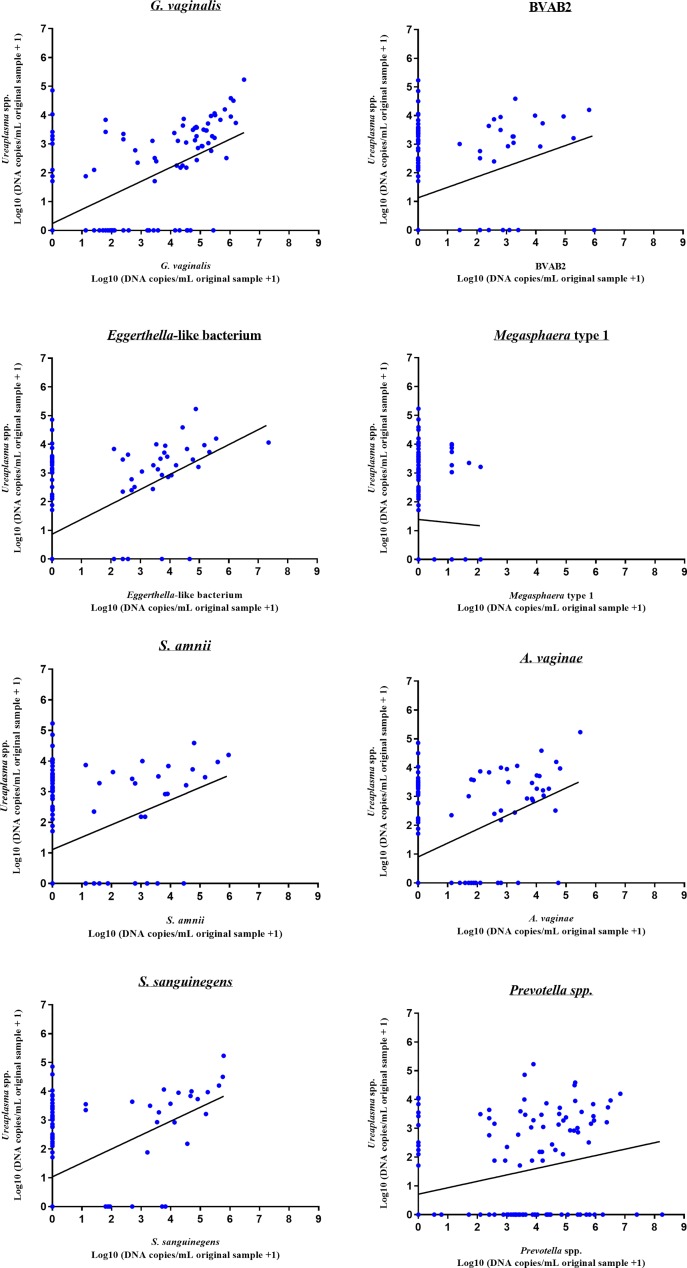
Comparison of the eight BV associated bacteria and total ureaplasma load (*U*. *urealyticum* and *U*. *parvum*) in all 141 samples. Regression lines are shown.

Just from looking at the unanalysed data, it was obvious that BV associated bacteria were present mainly in ureaplasma positive samples, and for both ureaplasma species, the bacterial load of all BV associated bacteria except *Megasphaera*-like type 1 were found to correlate with the ureaplasma load (data not shown). As it was speculated that the production of ammonia from urea by the ureaplasmas with the associated increase in pH could be the explanation for the increased bacterial load of BV associated bacteria, the combined ureaplasma DNA load (*U*. *urealyticum* plus *U*. *parvum*) was compared with the DNA load of each of the eight BV associated bacteria in all 141 samples (Fig 2). All BV associated bacteria, except *Megasphaera*-like type 1 (p = 0.63), were strongly positively correlated with the total ureaplasma load (p<0.0001–p<0.0005) using Spearman’s correlation coefficient. The strongest correlation was found for *G*. *vaginalis* (Rho = 0.60), the weakest for BVAB2 (Rho = 0.29).

The correlation between BV associated bacteria and ureaplasmas was weaker when the two ureaplasma species, *U*. *parvum* and *U*. *urealyticum* were compared separately.

## Discussion

In this cross-sectional case-control study, the role of eight BV associated bacteria in men with idiopathic urethritis (IU) was examined. *G*. *vaginalis*, BVAB-2, *Eggerthella*-like bacteria, *Megasphaera*-like type 1, *S*. *amnii*, *A*. *vaginae*, *S*. *sanguinegens* and *Prevotella* species were found equally often in men with IU and in controls. However, in a multivariate model adjusting for all tested pathogens, *Megasphaera*-like type 1 was associated with urethritis with an ORadj of 4.6, but the bacterial load of this species was very low and thus, the association is most likely not clinically relevant.

BV-associated species have been detected in penile skin, semen and urine specimens by others [10, 19, 30]. Although there is no strong evidence that male partners to women with BV should be prescribed antibiotics to prevent recurrence, a study by Liu *et al*. showed that the BV-like microbiota in the coronal sulcus skin specimens had a close match to the vaginal microbiota of their female partners [31]. The role of anaerobic bacteria as a cause of urethritis in men is still unclear. In this study only *Megasphaera*-like type 1 was associated with IU with an ORadj of 4.6 and there was a trend towards it being detected more often and in higher loads in IU than in controls although this did not reach statistical significance. *Megasphaera*-like type 1 is a recently discovered vaginal bacterium and has not yet been placed in a taxonomic group. It is susceptible to metronidazole and clindamycin in vitro [32]. Further studies are suggested to confirm the importance of this finding. In the present dataset, *Megasphaera*-like type 1 was present in a very low abundance in five of the 20 IU patients, thus, a cut-off such as has been suggested to increase the specificity of the detection of *U*. *urealyticum* [6] could not be calculated.

Manhart *et al*. found that *S*. *amnii* (previously *Leptotrichia amnionii*) and/or *S*. *sanguinegens* was more common in men with NGU than in controls [10], but this finding was not confirmed in the present study although the assay used in the present study did not cross-react with *S*. *sanguinegens*. Even if the sum of *S*. *amnii* and *S*. *sanguinegens* was used in the analysis, the two *Sneathia* species were neither more common nor present at higher loads in IU patients than in asymptomatic controls (p = 0.82 and 0.73, respectively). It is unclear, however, how the assay developed by Fredricks *et al*. [33] differs in the inclusivity of other *Sneathia* species as compared to the sum of the two species specific assays used in the present study. Similarly, we were not able to replicate our previous finding that *U*. *urealyticum* is associated with NMGNCNGU [6]. The reasons for these discrepancies are not clear, but our study was limited by the relatively small number of NMGNCNGU cases (n = 24) enrolled. In the present study, we further eliminated the *U*. *urealyticum* and virus positive patients to yield a strictly defined group of symptomatic IU, which limited the number of cases to 20. Actually, in order to obtain a power of 0.8 with a p-value of 0.05, an odds-ratio in univariate analysis should be as high as 10 in order to demonstrate a significant association with this number of cases and controls and this is a significant limitation of the study. On the other hand, none of the studied bacterial species, except for *Megaspaera*-like type I and *Prevotella* spp showed OR>1.5 in univariate analysis whereas several of the studied species showed ORs <1. Thus, expanding the study may not have changed the conclusions.

To our surprise, all BV associated bacteria, except *Megasphaera*-like type 1, were found to have an increasing bacterial load with increasing total ureaplasma load. This correlation existed even after including a subset of patients with urethritis caused by *C*. *trachomatis* or *M*. *genitalium*. The correlation was present for both ureaplasma species when analysed separately, but increased when the bacterial load from *U*. *parvum* and *U*. *urealyticum*, was summed. Other studies examining the female vaginal microbiota using sequencing and/or species specific PCRs have not shown this relationship [34]. This could possibly suggest a symbiotic relationship found only in the urethra and possibly just the male urethra. It could be speculated that the presence of ureaplasmas resulted in an elevated pH due to the release of ammonia from urea hydrolysis, the main energy-generating mechanism used in ureaplasmas. Similar to the suggestions that alkalinisation of vaginal fluid by menstruation and semen increases the growth of BV associated microbiota [35], the same pH change elicited by ureaplasmas in the urethra where an abundance of urea is available, could play a role. As the correlation between ureaplasma and BV associated bacteria was found unexpectedly a long time after sample collection, pH measurements of the urethral secretions were not possible. Nothing is known about the pH gradient along the urethra in urethritis patients and in healthy men, but with the development of pH micro-sensors it would be possible to study this in relation to the microbiota composition. The role of *U*. *urealyticum* in male NGU is still not settled, as studies have shown diverging results [5, 6, 36, 37] and the present study did not find any correlation.

The strength of the study is the broad testing for suspected NGU pathogens and the use of well-validated quantitative PCR assays for ureaplasmas and BV associated bacteria. A major limitation is the low number of IU cases examined and possibly the inclusion of asymptomatic controls attending for partner tracing. If BV was present in a large proportion of the female partners due to the strong association between chlamydia and BV, a possible difference may have been masked. In Sweden, contact tracing is mandatory according to the legislation. Usually, contact tracing reaches back one year, and thus, many partners had the contact before the index person acquired her chlamydial infection. One Swedish study reported that 35% of notified partners, where the outcome from testing was known were in fact *C*. *trachomatis* test negative [38], and all controls in the present study were *C*. *trachomatis* negative. That the status of the asymptomatic control may have some influence was suggested by a higher load of *G*. *vaginalis* in the 56 asymptomatic men attending for partner tracing as compared to the 41 men attending for an STI check-up (p = 0.02). However, using only the 41 men attending for check-up as controls for the 20 IU cases, did not change the lack of association between BV-associated bacteria and IU. Furthermore, the association between ureaplasma load and the load of BV-associated bacteria was independent of the urethritis status of the study subjects.

The ureaplasmas were not examined to serotype level, as this marker is not stably associated with the genetic relationship [39]

In summary BV associated bacteria were common in both men with and without urethritis and there was a strong correlation between these bacteria and the total ureaplasma load. These correlations are important to control for in studies of idiopathic urethritis. *Megasphaera*-like type 1 was associated with IU, but further studies are needed to confirm this finding.

## Supporting information

S1 Datafile Complete dataset with qPCR bacterial load and metadata. (XLSX)
